# Neoadjuvant Systemic Therapy for Breast Cancer: Factors Influencing Surgeons’ Referrals

**DOI:** 10.1245/s10434-016-5296-y

**Published:** 2016-06-09

**Authors:** Eleftherios Mamounas, Christine Poulos, Hans-Peter Goertz, Juan Marcos González, Amy Pugh, Vincent Antao

**Affiliations:** 1 University of Florida Health Cancer Center–Orlando Health, Orlando, FL USA; 2 RTI Health Solutions, Research Triangle Park, NC USA; 3 Genentech Inc., South San Francisco, CA USA

## Abstract

**Background:**

This study aimed to assess the influence of disease- and patient-related factors on surgeons’ decisions to refer patients with early-stage breast cancer (EBC) for neoadjuvant systemic therapy (NST).

**Methods:**

An online survey of United States surgeons evaluated the influence of selected disease- and patient-related factors on surgeons’ decisions, rated their influence (individually and in combination), and provided a relative ranking of jointly considered factors using best–worst scaling.

**Results:**

The participants in this study were 100 licensed surgeons. The surgeons referred approximately 25 % of EBC patients for NST to improve surgical management. Approximately 75 % of the surgeons agreed that NST is important for EBC, if only to improve surgical management. More than half were “very likely” to refer EBC patients for NST based on anatomicopathologic factors. Less than 50 % were “very likely” to do so when considering tumor phenotype factors. Tumor size and lymph node status were ranked highest in hypothetical patient scenarios. Regarding combinations of factors, the importance of any single factor varied according to the combinations presented. Less than half of the respondents were “very familiar,” and half were “somewhat familiar” with NST guidelines for breast cancer. More than half of the respondents were unaware that findings have shown achievement of pathologic complete response (pCR) after NST to be associated with improved survival.

**Conclusions:**

Surgeons’ decision to refer for NST is strongly driven by surgical management goals. Anatomicopathologic factors are more influential than tumor phenotype. However, no single disease or patient factor consistently drives the decision to refer for NST. Surgeons’ awareness of the association between pCR achievement and longer survival could be improved.

**Electronic supplementary material:**

The online version of this article (doi:10.1245/s10434-016-5296-y) contains supplementary material, which is available to authorized users.

The usual therapeutic approach for early-stage breast cancer (EBC) consists of surgical tumor resection followed by adjuvant systemic therapy with or without radiotherapy. For patients with locally advanced breast cancer, neoadjuvant systemic therapy (NST) is generally considered the standard of care, and for selected patients with operable breast cancer, NST has become an alternative to adjuvant chemotherapy.

In randomized clinical trials, neoadjuvant chemotherapy has shown equivalence to adjuvant chemotherapy in prolonging disease-free and overall survival.^1^–^3^ During the past decade, the addition of human epidermal growth factor receptor 2 (HER2)-targeted agents to neoadjuvant chemotherapy for patients with HER2-positive breast cancer has significantly increased pathologic complete response (pCR) rates, more than for other subtypes, and pCR has been associated with better outcomes.^4^–^6^ In particular, achievement of pCR was recently identified by the United States (U.S.) Food and Drug Administration (FDA) as a possible surrogate end point for accelerated approval of new drugs in EBC,^7^ and this finding was used to support the first accelerated approval of an anti-HER2 therapy (Perjeta [pertuzumab] in combination with trastuzumab and chemotherapy) for use in the neoadjuvant setting.^8^

NST has several potential clinical advantages including improved surgical options 2,3,9,10 and improved long-term outcomes.^2^,^10^,^11^ Also, clinicians have leveraged the ability to use NST to identify responders and nonresponders, offering the potential for further tailoring of systemic therapy options.^12^

This study aimed to assess the influence of disease- and patient-related factors on surgeons’ decisions to refer patients with EBC for NST.

## Methods

### Study Approach and Implementation

An ad hoc review of the literature showed that referrals for adjuvant therapy and its use for breast and other cancers are influenced by patient and tumor characteristics, the surgeon, and the care setting.^13^–^17^ However, less is known about factors that affect referral for NST and its use for breast cancer. This review informed identification of the following 11 disease and patient factors associated with referrals for NST:

- Skin/chest wall involvement
- Tumor size
- Histologic grade/type
- HER2 status
- Estrogen receptor (ER) and progesterone receptor (PR) status
- Inflammatory breast cancer (IBC)
- Involvement of axillary lymph nodes by clinical assessment (lymph node status)
- Patient’s age
- Patient’s overall health and comorbidities
- Patient’s preference for timing of surgery
- Patient’s level of interest in breast-conservation surgery.

Survey questions were developed to elicit the influence of selected individual and disease factors on surgeons’ decisions using the following three assessments:

- Ratings to determine the influence of all 11 individual disease and patient factors, independently, using a 4-point Likert scale (from “very likely” to “very unlikely” to refer for NST)
- Ratings to determine the influence of combinations of four disease factors (i.e., status of 2 tumor markers, lymph node involvement, and tumor size) using a 4-point Likert scale
- Relative ranking to determine the influence of multiple disease and patient factors when considered jointly.

The relative rankings were elicited using case 1 best–worst scaling (BWS).^18^,^19^ Surgeons were presented with three hypothetical and typical EBC patient scenarios, each defined in terms of nine disease and patient factors. For each scenario, surgeons determined whether they would first refer the patient for NST, refer the patient for adjuvant therapy, or proceed directly with surgery. In a series of BWS questions, each with three disease and patient factors determined by a predetermined experimental design with known statistical properties, the participating surgeons ranked the importance of each factor (e.g., 3-cm tumor, HER2-positive status, and grade 2 invasive ductal carcinoma) for each hypothetical patient.

The survey also included questions about respondents’ personal and practice characteristics. The survey was tested and refined based on in-person semistructured pretest interviews with a convenience sample of ten breast surgeons. The pretest participants confirmed that the list of disease and patient factors was comprehensive. The final survey is included as Supplementary Material.

 All Global (New York, NY), a vendor specializing in online surveys, programmed and hosted the online survey, with 100 surgeons recruited from a web panel of physicians. Physicians joined the panel via a double opt-in process. All Global verified the American Medical Association (AMA) or American Osteopathic Association (AOA) numbers of the panelists as well as their email and work addresses. The panelists were invited to participate in the survey via email. Respondents were required to be board-certified or board-eligible surgeons practicing in the United States who had performed breast cancer surgery for at least 2 years since completing surgical training and had completed at least 30 mastectomies or lumpectomies in the year before the survey.

The study was reviewed and approved by RTI International’s institutional review board.

### Statistical Analysis

The ratings of individual factors and combinations of factors were summarized by means and standard deviations. To analyze the BWS data, a logit model was used, following the methods presented by Yuan et al.,^20^ to infer the importance of disease and patient factors in the surgeons’ stated intention to refer hypothetical patients for one of three treatment options (NST, adjuvant therapy, surgery). One logit model was estimated for each hypothetical patient profile. The estimated parameter for a factor can be interpreted as the importance of that factor relative to the most important factor, which was normalized to 1.

## Results

All Global invited 1590 physicians in their U.S. web panel to be screened for study eligibility, and 483 responded to the invitation. Of those who responded, 117 were eligible to participate. All 117 eligible participants (100 %) were rescreened for specialty and consented to participate. Of these eligible physicians who consented to participate, 100 (85 %) completed the survey.

### Respondent Characteristics

In September 2014, 100 surgeons licensed in 46 states and the District of Columbia completed the survey. Approximately three-fourths (73 %) of the respondents were male. On the average, the respondents spent 74 % of each week in direct patient care. All the respondents had performed surgery significantly longer than 2 years (mean 17 ± 7 years) and had completed well over the required 30 lumpectomies or mastectomies in the previous year (mean 144 ± 149). Approximately one-half of the respondents were involved in private individual (21 %) or group (32 %) practices, and approximately one-fourth were involved in academic/university (18 %) or cancer center-based (9 %) practices. The remaining respondents were community hospital-based practitioners (19 %). General surgeons and surgical oncologists each comprised approximately one-third of the respondents (36 and 33 %, respectively). Breast surgeons comprised approximately one-fourth (26 %) of the respondents. A total of 76 surgeons indicated that they regularly presented at tumor boards and that 47 % of their patients, on the average, were presented with EBC.

### Identification of Aggressive Disease

**Fig. 1:**
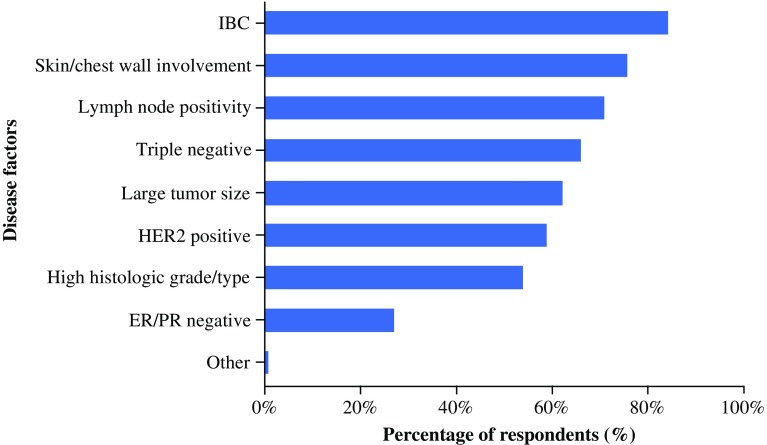
Disease factors each selected as one of five factors indicating tumor aggressiveness. *ER* estrogen receptor, *HER2* human epidermal growth factor receptor 2, *IBC* inflammatory breast cancer, *PR* progesterone receptor

From a list of disease factors provided, the respondents most frequently selected IBC and skin/chest wall involvement as two of the top five disease factors indicating tumor aggressiveness (Fig. 1). Additionally, other anatomicopathologic factors (lymph node positivity and, to some extent, tumor size) were selected more often than tumor phenotype factors (HER2 status, ER/PR status, and histologic grade/type), except for triple-negative breast cancer. However, more than half of the surgeons selected most of the phenotype factors (high histologic grade/histologic type, HER2-positive status, triple-negative status) as the top five indicators.

### Disease- and Patient-Related Factors for NST Referral

#### Ratings of Individual Factors

**Fig. 2:**
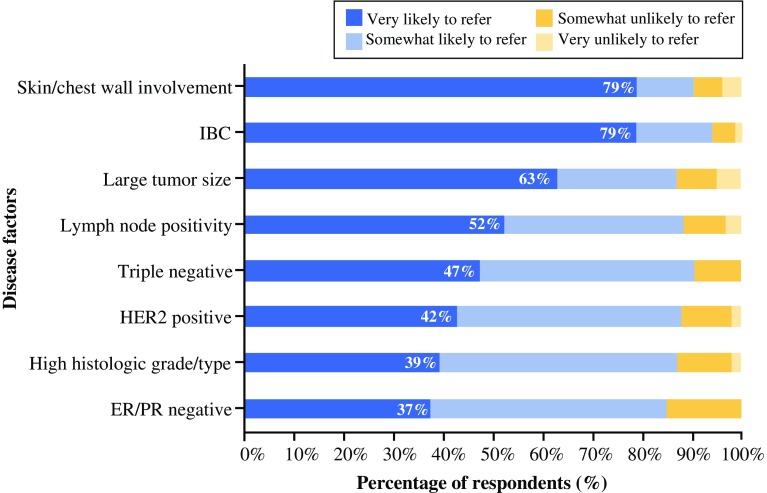
Ratings of likelihood that a patient will be referred for NST, by disease factor. *ER* estrogen receptor, *HER2* human epidermal growth factor receptor 2, *IBC* inflammatory breast cancer, *NST* neoadjuvant systemic therapy, *PR* progesterone receptor. *Note* Each respondent rated only the five characteristics that he or she associated the most with aggressiveness of disease

More than half of the respondents were “very likely” to refer a patient with EBC for NST based on each of the following anatomicopathologic factors of aggressiveness: lymph nodes (52 %), large tumor size (63 %), skin/chest wall involvement (79 %), and IBC (79 %) (Fig. 2). Less than half of respondents were “very likely” to refer a patient with EBC for NST based on the following tumor phenotype factors of aggressiveness: ER/PR-negative status (37 %), high histologic grade/histologic type (39 %), HER2-positive status (42 %), and triple-negative status (47 %). When the “very likely” and the “somewhat likely” ratings were combined, the similar total percentages indicated that all these anatomicopathologic and tumor phenotype factors were important in influencing a surgeon’s decision to refer for NST.

**Fig. 3:**
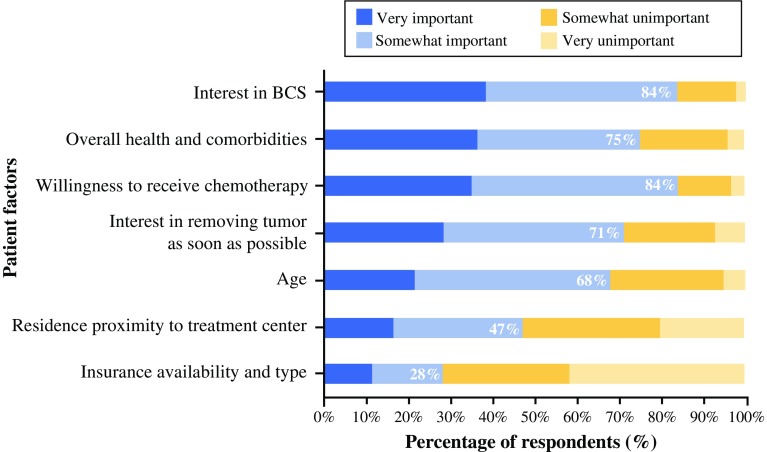
Ratings of the importance of individual patient factors and patient-related considerations in the decision to refer patients for consideration of NST. *BCS* breast cancer surgery, *NST* neoadjuvant systemic therapy

In addition to disease factors, the surgeons rated the influence that individual patient factors and patient-related considerations have on NST referrals (Fig. 3). The following factors were most commonly listed as “very important” or “somewhat important”: patient’s interest in breast-conservation surgery (84 %), tumor removal expediency (71 %), willingness to receive chemotherapy (84 %), overall health (75 %), and age (68 %). A lower percentage of surgeons reported practical concerns such as proximity to the treatment center (47 %) and insurance coverage (28 %) as “very important” or “somewhat important.”

#### Ratings for Combinations of Factors

**Fig. 4:**
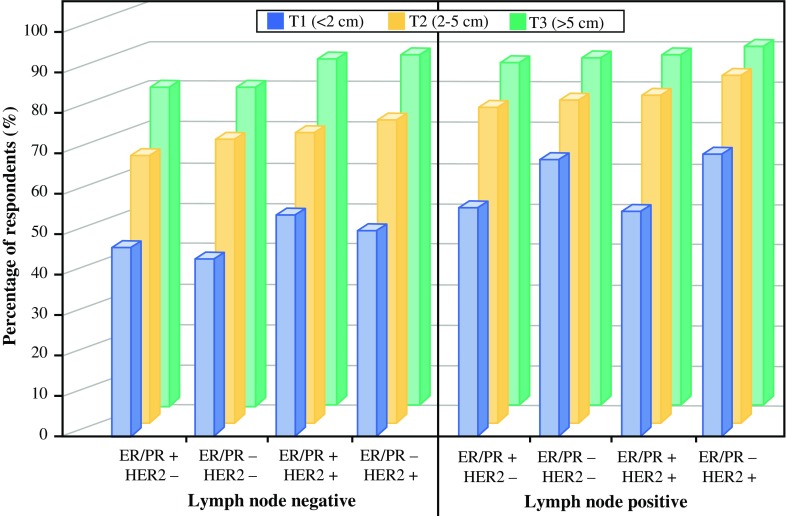
The percentage of respondents “somewhat likely” or “very likely” to refer a patient for NST, based on ER/PR status, HER2 status, and tumor size, by lymph node status. *ER* estrogen receptor, *HER2* human epidermal growth factor receptor 2, *NST* neoadjuvant systemic therapy, *PR* progesterone receptor

Responses to the questions about combinations of four factors (i.e., status of 2 tumor markers, lymph node involvement, and tumor size) indicated that given a tumor phenotype, the likelihood of referral for NST increased consistently with tumor size (T1 to T2 to T3) (Fig. 4). Lymph node status also appeared to increase the likelihood of referral for NST, but its relative influence decreased with tumor size. In general, differences in tumor phenotype did not significantly affect the likelihood of referral.

#### Rankings of Factors Considered Jointly in the Context of Patient Scenarios

**Fig. 5:**
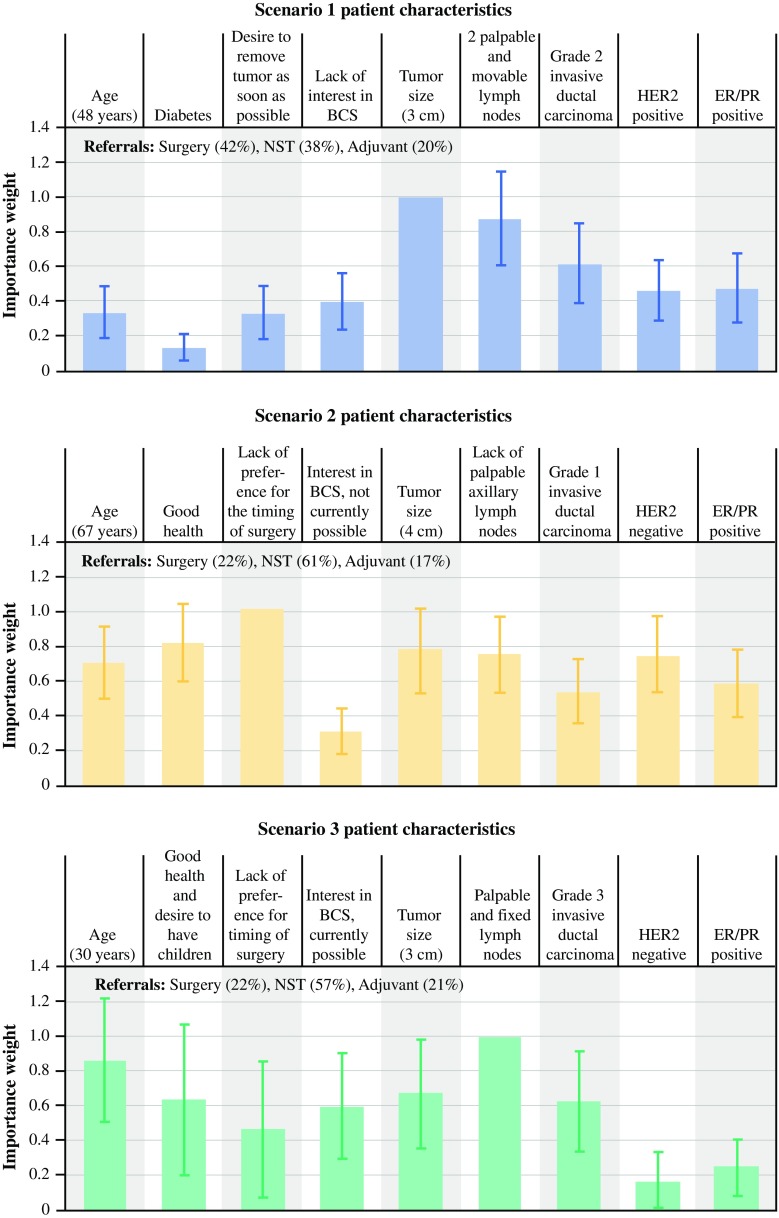
Relative ranking of factors by hypothetical patient scenario (*n* = 100). *CI* confidence interval. *Note* The *vertical bars* surrounding each mean preference weight denote the 95 % CI about the point estimate. “Tumor size,” “lack of preference for timing of surgery,” and “palpable and fixed lymph node” in patient scenarios 1, 2, and 3, respectively, do not have the 95 % CI because all the other factors were scaled relative to that factor

Figure 5 presents hypothetical patient scenarios and the within-scenario relative ranking of disease and patient factors in the decision to refer a patient for NST. The importance of the factors varied by scenario, but tumor size and lymph node status were the most highly rated disease factors for all three scenarios. The patient scenarios excluded IBC and skin/chest wall involvement, which would be expected to dominate the decision to refer for NST. Furthermore, although a patient’s willingness to receive chemotherapy ranked high in importance as an individual factor, we excluded it from BWS-ranking questions because we considered it an equally important factor for both neoadjuvant and adjuvant systemic therapy.

### Other Factors for NST Referral

#### Treatment Goals

On the average, the surgeons reported referring 48 % of patients with EBC to a medical oncologist before resection. Of these patients, 52 % were referred for NST to improve surgical management, whereas 25 % were referred to undergo NST for other reasons, and 21 % were referred for discussion of adjuvant therapy.

The majority of the surgeons (85 %) agreed that NST is an important part of treatment for stages 1, 2, or 3 breast cancer, even if only to improve surgical management so as to ensure negative margins, convert inoperable cases to operable cases, and/or convert mastectomy candidates to lumpectomy candidates.

The percentages of surgeons who responded that the following tumor-marker and other related information was “almost always” available to inform their decision to perform surgery ranged from 60 to 73 %, depending on the type of information, as follows: HER2 (61 %), ER/PR (60 %), type/grade (70 %), lymph node status (73 %), and tumor size (71 %). When this information was unavailable, the percentage of respondents who would “almost always” wait for HER2 status information before performing surgery was 33 % for stage 1 disease, 43 % for stage 2 disease, and 51 % for stage 3 disease.

#### Awareness of Evidence for NST Effectiveness

Most respondents (>90 %) reported some familiarity with specific NSTs for breast cancer, although less than half (40 %) were “very familiar” and approximately half (51 %) were “somewhat familiar” with NST therapies. Altogether, 96 % of the breast surgeons and 97 % of the surgical oncologists reported being “very familiar” (65 and 45 %, respectively) or “somewhat familiar” (31 and 52 %, respectively) with the NST therapies compared with 84 % of the general surgeons who rated themselves as “very familiar” (17 %) or “somewhat familiar” (67 %) with the NST therapies. More than half of the respondents were unaware of available evidence showing that patients who had a pCR after NST were more likely to have improved survival (event-free survival, disease-free survival, or overall survival). Only 54 % of the breast surgeons, 45 % of the surgical oncologists, and 31 % of the general surgeons reported awareness of this evidence.

For further assessment of familiarity with NSTs and evidence-supported use, the respondents were asked whether two scenarios might affect their decision to refer patients for NST. First, if an FDA-approved therapy showed a significant improvement in pCR rate, 39 % of the respondents were “very likely” and 48 % were “somewhat likely” to consider referral for NST therapy. Second, if an FDA-approved NST demonstrated significantly improved long-term efficacy, 52 % of the respondents were “very likely” and 36 % were “somewhat likely” to consider referral for NST.

## Discussion

To our knowledge, no previously published studies have investigated the stated importance of disease- and patient-related factors in surgeons’ decisions to refer patients for NST. Our survey showed that surgeons’ decisions to refer for NST are strongly driven by surgical management goals.

Based on the ratings of the individual disease factors assessed, IBC and skin/chest wall involvement were the most influential disease factors driving referrals for NST. Most patient factors and considerations also were rated highly, but these percentages were lower than those for the disease factors.

Overall, our survey found that anatomicopathologic factors are more influential than tumor phenotype in the decision to refer a patient for NST, suggesting that biologic factors are simply not as important in surgeons’ thinking as anatomicopathologic factors. However, the relative ranking of factors in the three patient scenarios (excluding IBC and skin/chest wall involvement) showed that no single disease or patient factor (of the 9 examined) consistently drives the decision to refer for NST. In fact, all the pre-identified factors provided in the survey were important in the decision to refer for NST, but which factor dominated depended on the other factors present.

Our survey results indicate that surgeons are at least somewhat aware of NSTs and their appropriate use. However, awareness among surgeons of the association between achievement of pCR and longer survival could be improved. A recent FDA-led meta-analysis 11 showed that pCR in breast and lymph nodes was associated with improved long-term survival compared with no pCR. Furthermore, Cortazar et al.^11^ found the strongest association between pCR and long-term survival among patients with aggressive breast cancer subtypes such as triple-negative status; ER/PR-positive status, HER2-negative status, and high-grade, and ER/PR-negative/HER2-positive disease status.

One interesting finding showed that tumor marker and other related information was “almost always” available to the surgeons in only 60–73 % of cases before surgery. Furthermore, when HER2 status was unavailable, the percentage of the surgeons who would “almost always” wait before performing surgery was only 33 % for stage 1 disease, only 43 % for stage 2 disease, and only 51 % for stage 3 disease. Although knowledge of tumor phenotype does not greatly alter the surgical resection plan, tumor size and nodal status clearly have important roles relative to the type of surgical approach for the breast and axilla. Moreover, knowledge of tumor phenotype is increasingly considered in the selection of appropriate candidates for NST. To that extent, educational efforts must focus on increasing the integration of tumor biomarkers in the treatment plan before surgery so as not to deprive appropriate candidates for NST from the opportunity to receive it.

Although the pre-identified nature of the factors rated or ranked by the surgeons was a possible limitation of the study, the factors selected were informed by an ad hoc review of the literature and pretested in interviews. Another potential limitation of such a survey is that sample representativeness of the study respondents and findings cannot be determined. Physicians opted in both to the panel from which they were recruited and to survey participation, which potentially influenced sample representativeness. Sample characteristics were not compared with those of surgeons treating breast cancer in the United States. In addition, the questions included in the survey and the conclusions drawn from the surgeons’ responses to these questions represent an exploration into surgeons’ practices for the referral of breast cancer patients for NST. Given this objective and the nature of the questions considered in the survey, the data would not support meaningful formal statistical inferences based on the patterns of the surgeons’ responses. Nevertheless, our results can inform the design of future studies, which will allow meaningful statistical tests around the issues we explored.

In summary, surgeons’ decisions to refer patients for NST are made primarily to improve surgical outcomes. Anatomicopathologic factors influence referral decisions more than tumor phenotype factors, but although these factors are important, no single pre-identified factor drives the NST referral decision. Finally, less than half of surgeons responding were aware of the correlation between pCR achievement and long-term survival, indicating that this awareness could be improved and NST referral possibly considered more widely for patients with EBC.

## Electronic supplementary material

Below is the link to the electronic supplementary material.

Supplementary material 1 (DOCX 63 kb)
