# Supplementary material for: Neoadjuvant Systemic Therapy for Breast Cancer: Factors Influencing Surgeons’ Referrals
Source: Ann Surg Oncol. 2016 Jun 9;23(11):3510–7. doi: 10.1245/s10434-016-5296-y (PMC5009159; doi:10.1245/s10434-016-5296-y)
Supplement: Supplementary file 1 — Supplementary material 1 (DOCX 63 kb) [file 10434_2016_5296_MOESM1_ESM.docx]

# supplementary appendix

Final Study Survey

[Screening Questions]

1. Are you 18 years of age or older?

- Yes [Continue]
- No [Ineligible, end survey]

1. Are you a board-certified or board-eligible surgeon practicing in the United States?

- Yes [Continue]
- No [Ineligible, end survey]

1. In the past year, approximately how many mastectomies or lumpectomies have you completed to remove the primary breast tumor in stage 1-3 breast cancer patients?

____________ [RANGE 0-999]

[If equal or greater than 30, continue. If less than 30, ineligible -- end survey.]

1. How many years have you been performing breast cancer surgery since completing your surgical training?

____________years [RANGE 0-70]

[If at least 2 years, continue. If less than 2 years, ineligible -- end survey. If greater than 35 years, ineligible – end survey.]

1. Which best characterizes the hospital with which you are primarily affiliated?

- Community – Non-Teaching
- Community – Teaching
- Academic/University

[Our goal is to recruit between 25% to 40% for EACH of these three groups. SET UP MAX 40 FOR EACH]

## Study Purpose

You are one of about 100 surgeons who are being asked to take this survey to help us understand factors that affect decisions about treating early breast cancer.

## Study Duration

The survey will take about 20 minutes to complete.

## Study Details

Research Triangle Institute (RTI) is doing this study for a pharmaceutical company. RTI is a non-profit research organization in Research Triangle Park, North Carolina.

## Possible Risks or Discomforts

RTI will protect your responses under its Privacy Policy. RTI will also make every effort to protect your responses. There is a potential risk of disclosure of the survey data, but the data could not be directly tied to you.

## Benefits

There are no direct benefits to you for participating in this study. Benefits of this survey apply to the broader population such that it will generate a better understanding of factors that affect decisions about treating early breast cancer.

## Confidentiality

Many steps have been taken to protect your information. RTI will report only your responses to the survey, not your name or other contact information. If the results of this study are presented at scientific meetings or published in scientific journals, no information will be included that could identify you or your responses personally.

The Institutional Review Board (IRB) at RTI has reviewed this research. An IRB is a group of people who make sure that the rights of participants in research are protected. The IRB may check records of your activity in this research to see if proper procedures were followed.

## Your Rights

Your decision to take part in this research study is completely voluntary.

If you have any questions about your rights as a participant of this study, please send an email to the RTI Office of Research Protection at [orpe@rti.org](mailto:orpe@rti.org).

Referral Patterns for Stage 1-3 Breast Cancer

1. In the last 6 months, about what percentage of your stage 1-3 breast cancer patients did you refer to a medical oncologist ***PRIOR*** to resecting the primary tumor?

_________ % [RANGE 0-100]

ASKED IF Q1>0

1. Of these stage 1-3 breast cancer patients that you referred to a medical oncologist ***PRIOR*** to surgery, what percentages were for the following reasons?

*Responses should sum to 100%.*

| **Main reason for referring stage 1-3 breast cancer patients to medical oncologist *PRIOR* to resecting the primary tumor** | **Percentage of stage 1-3 breast cancer patients** |
| --- | --- |
| Consideration of neoadjuvant systemic therapy to improve surgical management (eg. ensure negative margins, convert inoperable cases to operable cases, convert mastectomy candidates to lumpectomy candidates, convert axillary lymph node dissection cases to sentinel lymph node dissection cases, reduce the extent of radiotherapy, etc.) | ______ % |
| Consideration of neoadjuvant systemic therapy for reasons other than to improve surgical management (e.g. identify patients with better/worse prognosis based on response, improve long-term outcomes, etc.) | ______ % |
| Consideration of adjuvant systemic therapy only | ______ % |
| Other | ______ % |

Total ______%

1. About how frequently do you interact with a medical oncologist (either one-on-one or as part of a group, such as a tumor board) about the following topics, either in general or with respect to a particular case:

GRID WITH SCALE AT THE TOP

| **Topics** | **Frequency** |
| --- | --- |
| Neoadjuvant systemic therapy to improve surgical management | - Weekly - Monthly - Quarterly - Annually - Almost never / Never |
| Neoadjuvant systemic therapy for reasons other than surgical management | - Weekly - Monthly - Quarterly - Annually - Almost never / Never |
| Adjuvant systemic therapy | - Weekly - Monthly - Quarterly - Annually - Almost never / Never |

1. Which of the following statements best describes you?

- I am very familiar with ***specific*** neoadjuvant systemic therapies for breast cancer
- I am somewhat familiar with ***specific*** neoadjuvant systemic therapies for breast cancer
- I am not familiar with ***specific*** neoadjuvant systemic therapies for breast cancer

1. In general, how often is information on the following prognostic markers available to you at the time you make a decision to perform surgery?

| **Prognostic marker** | **Almost always available** | **Usually available** | **Usually not available** | **Almost never available** |
| --- | --- | --- | --- | --- |
| HER2 status | □ | □ | □ | □ |
| ER/PR status | □ | □ | □ | □ |
| Histological type/grade | □ | □ | □ | □ |
| Clinical assessment of lymph node status | □ | □ | □ | □ |
| Tumor size | □ | □ | □ | □ |

1. How likely are you to wait for information on HER2 status to perform surgery on patients in each of the following stages:

| **Stage** | **Almost always** | **Usually** | **Usually not** | **Almost never** |
| --- | --- | --- | --- | --- |
| Stage 1 | □ | □ | □ | □ |
| Stage 2 | □ | □ | □ | □ |
| Stage 3 | □ | □ | □ | □ |

1. Do you believe neoadjuvant systemic therapy followed by adjuvant therapy provides additional long-term efficacy benefit over adjuvant therapy alone?

- Yes
- No

1. How likely are the following factors to influence your decision to refer patients with stage 1-3 breast cancer to a medical oncologist for neoadjuvant systemic therapy:

| **Factors** | **Very Likely** | **Somewhat Likely** | **Somewhat Unlikely** | **Very Unlikely** |
| --- | --- | --- | --- | --- |
| Concerns about delaying surgery due to adverse events resulting from neoadjuvant systemic therapy | □ | □ | □ | □ |
| Concerns about inaccurately staging the axilla following neoadjuvant systemic therapy | □ | □ | □ | □ |
| Concerns about delaying surgery due to time for full course of neoadjuvant systemic therapy | □ | □ | □ | □ |

**Patient Vignettes**

Suppose you were treating the following patient:

**Patient #1:**

- 48-year old female with diabetes
- 3-cm ER/PR-positive and HER2-positive breast cancer
- 2 palpable and movable lymph nodes (1.5 - 2.0 cm) in the left axilla
- Grade 2 invasive ductal carcinoma
- Patient wants to remove the tumor as soon as possible
- Patient does not have a desire for breast conservation surgery (BCS)
- Breast/tumor ratio allows for BCS

1. Which of the following would you do first for Patient #1?

- Refer this patient to a medical oncologist for consideration for neoadjuvant systemic therapy (may include endocrine therapy)
- Refer this patient to a medical oncologist to discuss adjuvant systemic therapy options
- Perform surgery

You said that the first thing you would do for Patient #1 is:

- [Refer this patient to a medical oncologist for consideration for neoadjuvant systemic therapy
- Refer this patient to a medical oncologist to discuss adjuvant systemic therapy
- Perform surgery]

1. Which of the 3 factors shown below would be most important and least important in your decision about what to do first for Patient #1?

| **Most important factor in decision** | **Possible factors influencing decision**  **BASED ON DESIGN** | **Least important factor in decision** |
| --- | --- | --- |
| □ | Size of patient’s tumor (3 cm) | □ |
| □ | HER2 positive status | □ |
| □ | Patient’s age (48 years) | □ |

1. Which of the 3 factors shown below would be most important and least important in your decision about what to do first for Patient #1?

| **Most important factor in decision** | **Possible factors influencing decision**  **BASED ON DESIGN** | **Least important factor in decision** |
| --- | --- | --- |
| □ | Patient’s histological grade/histology type: Grade 2 invasive ductal carcinoma | □ |
| □ | Patient has diabetes | □ |
| □ | Patient’s lack of interest in breast conservation surgery | □ |

1. Which of the 3 factors shown below would be most important and least important in your decision about what to do first for Patient #1?

| **Most important factor in decision** | **Possible factors influencing decision**  **BASED ON DESIGN** | **Least important factor in decision** |
| --- | --- | --- |
| □ | Size of patient’s tumor (3 cm) | □ |
| □ | Patient’s ER/PR positive status | □ |
| □ | Patient’s histological grade/histology type: Grade 2 invasive ductal carcinoma | □ |

1. Which of the 3 factors shown below would be most important and least important in your decision about what to do first for Patient #1?

| **Most important factor in decision** | **Possible factors influencing decision**  **BASED ON DESIGN** | **Least important factor in decision** |
| --- | --- | --- |
| □ | Size of patient’s tumor (3 cm) | □ |
| □ | HER2 positive status | □ |
| □ | Patient’s age (48 years) | □ |

Now suppose you were treating a different patient with the following characteristics:

**Patient #2:**

- 67-year old female in good health
- Operable 4-cm ER/PR-positive and HER2-negative breast cancer
- No palpable axillary lymph nodes
- Grade 1 invasive ductal carcinoma
- Patient has no preference for timing of surgery
- Patient has an interest in breast conservation surgery
- Breast/tumor ratio does not currently allow for BCS

1. Which of the following would you do first for Patient #2?

- Refer this patient to a medical oncologist for consideration for neoadjuvant systemic therapy (may include endocrine therapy)
- Refer this patient to a medical oncologist for consideration for adjuvant systemic therapy options
- Perform surgery

You said that first thing you would do for Patient #2 is

- [Refer this patient to a medical oncologist for consideration for neoadjuvant systemic therapy
- Refer this patient to a medical oncologist to discuss adjuvant systemic therapy
- Perform surgery]

1. Which of the 3 factors shown below would be most important and least important in your decision about what to do first for Patient #2?

| **Most important factor in decision** | **Possible factors influencing decision**  **BASED ON DESIGN** | **Least important factor in decision** |
| --- | --- | --- |
| □ | Patient has no preference for timing of surgery | □ |
| □ | Patient’s age (67 years) | □ |
| □ | Patient’s good health | □ |

1. Which of the 3 factors shown below would be most important and least important in your decision about what to do first for Patient #2?

| **Most important factor in decision** | **Possible factors influencing decision**  **BASED ON DESIGN** | **Least important factor in decision** |
| --- | --- | --- |
| □ | Patient’s HER2-negative status | □ |
| □ | Patient’s good health | □ |
| □ | Size of patient’s tumor (4 cm) | □ |

1. Which of the 3 factors shown below would be most important and least important in your decision about what to do first for Patient #2?

| **Most important factor in decision** | **Possible factors influencing decision**  **BASED ON DESIGN** | **Least important factor in decision** |
| --- | --- | --- |
| □ | Patient's lack of preference for the timing of surgery | □ |
| □ | Patient’s HER2-negative status | □ |
| □ | Patient’s ER/PR positive status | □ |

1. Which of the 3 factors shown below would be most important and least important in your decision about what to do first for Patient #2?

| **Most important factor in decision** | **Possible factors influencing decision**  **BASED ON DESIGN** | **Least important factor in decision** |
| --- | --- | --- |
| □ | Size of patient’s tumor (4 cm) | □ |
| □ | Patient’s HER2-negative status | □ |
| □ | Lack of palpable axillary lymph nodes | □ |

Now suppose you were treating a different patient with the following characteristics:

**Patient #3:**

- 30-year old female in good health, desires to have children
- 3-cm ER/PR-negative and HER2-positive breast cancer
- 1 cm palpable and fixed lymph node in the left axilla
- Grade 3 invasive ductal carcinoma
- Patient has no preference for timing of surgery
- Patient has an interest in breast conservation surgery
- Breast/tumor ratio allows for BCS

1. Which of the following would you do first for Patient #3?

- Refer this patient to a medical oncologist for consideration for neoadjuvant systemic therapy
- Refer this patient to a medical oncologist for consideration for adjuvant systemic therapy options
- Perform surgery

1. You said that first thing you would do for Patient #3 is

- [Refer this patient to a medical oncologist for consideration for neoadjuvant systemic therapy
- Refer this patient to a medical oncologist to discuss adjuvant systemic therapy
- Perform surgery]

Which of the 3 factors shown below would be most important and least important in your decision about what to do first for Patient #3?

| **Most important factor in decision** | **Possible factors influencing decision**  **BASED ON DESIGN** | **Least important factor in decision** |
| --- | --- | --- |
| □ | Patient’s HER2-negative status | □ |
| □ | Patient's interest in breast conservation surgery | □ |
| □ | Patient’s ER/PR negative status | □ |

1. Which of the 3 factors shown below would be most important and least important in your decision about what to do first for Patient #3?

| **Most important factor in decision** | **Possible factors influencing decision**  **BASED ON DESIGN** | **Least important factor in decision** |
| --- | --- | --- |
| □ | Patient’s age (30 years) | □ |
| □ | Patient’s palpable and fixed lymph node | □ |
| □ | Patient’s ER/PR negative status | □ |

1. Which of the 3 factors shown below would be most important and least important in your decision about what to do first for Patient #3?

| **Most important factor in decision** | **Possible factors influencing decision**  **BASED ON DESIGN** | **Least important factor in decision** |
| --- | --- | --- |
| □ | Patient’s palpable and fixed lymph node | □ |
| □ | Patient's lack of preference for timing of surgery | □ |
| □ | Histological grade/histology type: Grade 3 invasive ductal carcinoma | □ |

1. Which of the 3 factors shown below would be most important and least important in your decision about what to do first for Patient #3?

| **Most important factor in decision** | **Possible factors influencing decision**  **BASED ON DESIGN** | **Least important factor in decision** |
| --- | --- | --- |
| □ | Patient’s good health and desire to have children | □ |
| □ | Patient's lack of preference for timing of surgery | □ |
| □ | Patient’s HER2-positive status | □ |

Questions about neoadjuvant systemic therapy

1. Please think about a case with the following prognostic markers:

**Case A**

- - HER2 positive
  - ER/PR positive
  - Lymph node positive, by clinical assessment

How likely would you be to refer this case to a medical oncologist for consideration of neoadjuvant systemic therapy if their tumor size was one of the following? (Assume average breast size.)

|  | **Very likely to refer** | **Somewhat likely to refer** | **Somewhat unlikely to refer** | **Very unlikely to refer** |
| --- | --- | --- | --- | --- |
| Tumor < 2 cm | □ | □ | □ | □ |
| Tumor 2-5 cm | □ | □ | □ | □ |
| Tumor > 5 cm | □ | □ | □ | □ |

1. Please think about a different case now, with the following prognostic markers:

**Case B**

- - HER2 negative
  - ER/PR negative
  - Lymph node negative, by clinical assessment

How likely would you be to refer this case to a medical oncologist for consideration of neoadjuvant systemic therapy? (Assume average breast size.)

|  | **Very likely to refer** | **Somewhat likely to refer** | **Somewhat unlikely to refer** | **Very unlikely to refer** |
| --- | --- | --- | --- | --- |
| Tumor < 2 cm | □ | □ | □ | □ |
| Tumor 2-5 cm | □ | □ | □ | □ |
| Tumor > 5 cm | □ | □ | □ | □ |

**Case C**

- - HER2 positive
  - ER/PR negative
  - Lymph node positive, by clinical assessment

How likely would you be to refer this case to a medical oncologist for consideration of neoadjuvant systemic therapy? (Assume average breast size.)

|  | **Very likely to refer** | **Somewhat likely to refer** | **Somewhat unlikely to refer** | **Very unlikely to refer** |
| --- | --- | --- | --- | --- |
| Tumor < 2 cm | □ | □ | □ | □ |
| Tumor 2-5 cm | □ | □ | □ | □ |
| Tumor > 5 cm | □ | □ | □ | □ |

**Case D**

- - HER2 negative
  - ER/PR positive
  - Lymph node positive, by clinical assessment

How likely would you be to refer this case to a medical oncologist for consideration of neoadjuvant systemic therapy? (Assume average breast size.)

|  | **Very likely to refer** | **Somewhat likely to refer** | **Somewhat unlikely to refer** | **Very unlikely to refer** |
| --- | --- | --- | --- | --- |
| Tumor < 2 cm | □ | □ | □ | □ |
| Tumor 2-5 cm | □ | □ | □ | □ |
| Tumor > 5 cm | □ | □ | □ | □ |

**Case E**

- - HER2 positive
  - ER/PR positive
  - Lymph node negative, by clinical assessment

How likely would you be to refer this case to a medical oncologist for consideration of neoadjuvant systemic therapy? (Assume average breast size.)

|  | **Very likely to refer** | **Somewhat likely to refer** | **Somewhat unlikely to refer** | **Very unlikely to refer** |
| --- | --- | --- | --- | --- |
| Tumor < 2 cm | □ | □ | □ | □ |
| Tumor 2-5 cm | □ | □ | □ | □ |
| Tumor > 5 cm | □ | □ | □ | □ |

**Case F**

- - HER2 negative
  - ER/PR positive
  - Lymph node negative, by clinical assessment

How likely would you be to refer this case to a medical oncologist for consideration of neoadjuvant systemic therapy? (Assume average breast size.)

|  | **Very likely to refer** | **Somewhat likely to refer** | **Somewhat unlikely to refer** | **Very unlikely to refer** |
| --- | --- | --- | --- | --- |
| Tumor < 2 cm | □ | □ | □ | □ |
| Tumor 2-5 cm | □ | □ | □ | □ |
| Tumor > 5 cm | □ | □ | □ | □ |

**Case G**

- - HER2 negative
  - ER/PR negative
  - Lymph node positive, by clinical assessment

How likely would you be to refer this case to a medical oncologist for consideration of neoadjuvant systemic therapy? (Assume average breast size.)

|  | **Very likely to refer** | **Somewhat likely to refer** | **Somewhat unlikely to refer** | **Very unlikely to refer** |
| --- | --- | --- | --- | --- |
| Tumor < 2 cm | □ | □ | □ | □ |
| Tumor 2-5 cm | □ | □ | □ | □ |
| Tumor > 5 cm | □ | □ | □ | □ |

**Case H**

- - HER2 positive
  - ER/PR negative
  - Lymph node negative, by clinical assessment

How likely would you be to refer this case to a medical oncologist for consideration of neoadjuvant systemic therapy? (Assume average breast size.)

|  | **Very likely to refer** | **Somewhat likely to refer** | **Somewhat unlikely to refer** | **Very unlikely to refer** |
| --- | --- | --- | --- | --- |
| Tumor < 2 cm | □ | □ | □ | □ |
| Tumor 2-5 cm | □ | □ | □ | □ |
| Tumor > 5 cm | □ | □ | □ | □ |

1. Choose the five disease characteristics you associate most with the ***aggressiveness*** of disease in stage 1-3 breast cancer.

*Choose 5 responses.*

- Skin/chest wall involvement
- Large tumor size
- High histologic grade / histologic type
- HER2-positive
- ER/PR-negative
- Triple-negative
- Inflammatory breast cancer
- Involved axillary lymph nodes by clinical assessment
- Other

ASK IF Q32 ANSWERED

1. Please indicate how likely it is that you refer a patient with each of the following disease characteristics to neoadjuvant therapy. (*Check all that apply.*)

|  | **Very likely to refer** | **Somewhat likely to refer** | **Somewhat unlikely to refer** | **Very unlikely to refer** |
| --- | --- | --- | --- | --- |
| [Factor #1 selected above] | □ | □ | □ | □ |
| [Factor #2 selected above] | □ | □ | □ | □ |
| [Factor #3 selected above] | □ | □ | □ | □ |
| [Factor #4 selected above] | □ | □ | □ | □ |
| [Factor #5 selected above] | □ | □ | □ | □ |

Programmers: Please use the following labels for the factors for the question above.

- Disease with skin/chest wall involvement
- Large tumor size
- Disease with high histologic grade / histologic type
- HER2-positive disease
- ER/PR-negative disease
- Triple-negative disease
- Inflammatory breast cancer
- Disease with involved axillary lymph nodes by clinical assessment

1. In the past 6 months, how important have the following patient-related factors been in your decision to either refer or not refer patients with stage 1-3 breast cancer to a medical oncologist for consideration of neoadjuvant systemic therapy?

| **Patient-related factors** | **Very Important** | **Somewhat Important** | **Somewhat Unimportant** | **Very Unimportant** |
| --- | --- | --- | --- | --- |
| Patient’s age | □ | □ | □ | □ |
| Patient’s overall health and comorbidities | □ | □ | □ | □ |
| Patient’s insurance provider (or lack of insurance) | □ | □ | □ | □ |
| Patient’s willingness (or unwillingness) to receive chemotherapy and/or targeted therapy | □ | □ | □ | □ |
| Patient’s level of interest in removing tumor as soon as possible | □ | □ | □ | □ |
| Patient’s level of interest in breast conservation surgery | □ | □ | □ | □ |
| Patient’s residential location or distance from treatment center | □ | □ | □ | □ |

1. Have you ever referred a patient to a medical oncologist for consideration for neoadjuvant systemic therapy to only acquire supplemental prognostic information and/or to tailor systemic therapy options (e.g., response-guided therapy)?

- Yes
- No

1. [If yes to Q35] How important was this reason when deciding to make these referrals?

- Very important
- Somewhat important
- Somewhat unimportant
- Very unimportant

1. Indicate your level of agreement with the following statement: Neoadjuvant systemic therapy is an important part of stage 1-3 breast cancer treatment only to improve surgical management (eg. ensure negative margins, convert inoperable cases to operable cases, convert mastectomy candidates to lumpectomy candidates, convert axillary lymph node dissection cases to sentinel lymph node dissection cases, reduce the extent of radiotherapy, etc.).

- Strongly agree
- Agree
- Disagree
- Strongly disagree

1. Do you or your patients have difficulty arranging a timely patient appointment when making referrals to consult with a medical oncologist for neoadjuvant or adjuvant systemic therapy?

- Yes
- No
- Don’t know
- Not applicable to my practice

1. How often have patients you referred for neoadjuvant systemic therapy been sent to another surgeon, and not returned to you, for surgery?

- Never
- Occasionally
- Frequently

1. Indicate your level of agreement with the following statements:

| **Statements** | **Strongly Agree** | **Agree** | **Disagree** | **Strongly Disagree** |
| --- | --- | --- | --- | --- |
| I am familiar with the clinical practice guidelines for neoadjuvant systemic therapy for breast cancer | □ | □ | □ | □ |
| I find the guidelines clear as to when neoadjuvant systemic therapy for breast cancer is recommended | □ | □ | □ | □ |
| I believe there is adequate scientific evidence to support current clinical practice guidelines regarding neoadjuvant systemic therapy | □ | □ | □ | □ |
| When used according to clinical practice guidelines, currently-available neoadjuvant systemic therapies are very effective in achieving pathological complete response (pCR) | □ | □ | □ | □ |

1. Which clinical practice guidelines do you follow? (*Check all that apply*)

- National Comprehensive Cancer Network (NCCN) guidelines
- American Society for Breast Surgeons (ASBS)
- American College of Surgeons (ACS)
- The hospital where I practice
- The hospital where I trained
- Other

1. Suppose a systemic therapy approved by the FDA also shows a significant improvement in pathological complete response (pCR) rate. How likely is this information to affect your decision to refer patients for neoadjuvant therapy that includes this therapy?

- Very likely
- Somewhat likely
- No effect
- Somewhat unlikely
- Very unlikely

1. Suppose this systemic therapy was only available in the neoadjuvant setting. How likely would you then be to refer patients for neoadjuvant systemic therapy that includes this therapy?

- Very likely
- Somewhat likely
- No effect
- Somewhat unlikely
- Very unlikely

1. Suppose a new neoadjuvant systemic therapy approved by the FDA demonstrated significant improvement in long-term efficacy (EFS/DFS and OS) in stage 1-3 breast cancer. How likely would this information be to affect your decision to refer patients for neoadjuvant systemic therapy that includes this therapy? (EFS=Event free survival, DFS=Disease free survival, OS=Overall survival)

- Very likely
- Somewhat likely
- No effect
- Somewhat unlikely
- Very unlikely

1. Are you aware of any publications of clinical studies or meta-analyses that showed patients who had a pathological complete response (pCR) after systemic neoadjuvant therapy were more likely to have improved survival (EFS/DFS and OS)?

- Yes
- No

Questions about you and your practice

1. What is your gender?

- Female
- Male

B2a In how many states are you licensed to practice?

______

B2 In which state or states are you licensed to practice?

____________________ 50 US STATES + DC

B3. What is your primary specialty?

- Breast surgery
- General surgery
- Surgical oncology
- Plastic surgery
- Other

B4. Which of the following best describes your principal practice setting (i.e., the practice in which you spend the majority of your time)?

- Private individual practice
- Private group practice
- Academic, University-based practice
- Cancer center-based practice
- Veteran’s hospital-based practice
- Community teaching hospital-based practice
- Community non-teaching hospital-based practice
- Other

B5. Do you regularly discuss your patients at the multi-disciplinary tumor board?

- Yes
- No

B6. [If response to B5 is “Yes”] What percentage of your stage 1-3 breast cancer patients are discussed at tumor boards prior to the decision to go to surgery?

________________% RANGE 0-100

B7. What percentage of your time is spent in providing direct patient care during a typical week? Direct patient care includes: seeing patients (including attending), reviewing tests, preparing for and performing surgery/procedures, providing other related patient care services (for example, communicating with the patient and/or family members and other professionals, including written and telephone reports). Do not include standby time, on-call time, or travel time when not seeing patients.

_______ Percentage of hours of direct patient care during a typical week RANGE 0-100

B8. Approximately what percentages of your breast cancer patients at your principal practice setting have the following primary source of payment? *(Responses should total to 100%)*

Medicare: ____ %

Medicaid: ____%

Self-pay, including discounted care for under/uninsured: ____%

Private Insurance and all other: _____%

Total ________% [add percentages from rows above]
